# Boron Delivery to Brain Cells via Cerebrospinal Fluid (CSF) Circulation for BNCT in a Rat Melanoma Model

**DOI:** 10.3390/biology11030397

**Published:** 2022-03-03

**Authors:** Sachie Kusaka, Yuri Morizane, Yugo Tokumaru, Shingo Tamaki, Indah Rosidah Maemunah, Yoko Akiyama, Fuminobu Sato, Isao Murata

**Affiliations:** Division of Sustainable Energy and Environmental Engineering, Graduate School of Engineering, Osaka University, Yamadaoka 2-1, Suita 565-0871, Osaka, Japan; yuri1154.sg@gmail.com (Y.M.); tokumaru@qb.see.eng.osaka-u.ac.jp (Y.T.); tamaki@see.eng.osaka-u.ac.jp (S.T.); indahrm@qr.see.eng.osaka-u.ac.jp (I.R.M.); yoko-ak@see.eng.osaka-u.ac.jp (Y.A.); fsato@see.eng.osaka-u.ac.jp (F.S.); murata@see.eng.osaka-u.ac.jp (I.M.)

**Keywords:** boron neutron capture therapy (BNCT), cerebrospinal fluid (CSF), boron delivery, brain tumor, melanoma model rat, blood–brain barrier (BBB)

## Abstract

**Simple Summary:**

The blood–brain barrier (BBB) is formed by the brain capillary endothelium and prevents almost all therapeutic agents from reaching the brain. The importance of the BBB in brain tumor treatments has not been recognized until recently, including in the case of boron neutron capture therapy (BNCT), although it affects therapeutic efficacy when treating brain tumors. Recently, some drug delivery systems to bypass the BBB have been developed for brain tumor therapy, and our laboratory has been developing a system for boron delivery to brain cells using cerebrospinal fluid (CSF) circulation, which we call the “boron CSF administration method”. In this study, we carried out experiments with brain tumor model rats to demonstrate the usefulness of the CSF administration method for BNCT. As a result, we found that boron injected using the CSF administration method accumulates to high levels in tumor cells, with a high T/N ratio. In addition, the dose required for the boron drug was much lower than that used in the intravenous (IV) administration method for equivalent effects. This approach makes it possible for clinicians to inject a lower drug dose into patient, thus reducing the potential side effects of excessive amounts of the drug and decreasing its cost. We hope our findings will inspire additional studies on boron delivery to brain tumors for BNCT.

**Abstract:**

Recently, exploitation of cerebrospinal fluid (CSF) circulation has become increasingly recognized as a feasible strategy to solve the challenges involved in drug delivery for treating brain tumors. Boron neutron capture therapy (BNCT) also faces challenges associated with the development of an efficient delivery system for boron, especially to brain tumors. Our laboratory has been developing a system for boron delivery to brain cells using CSF, which we call the “boron CSF administration method”. In our previous study, we found that boron was efficiently delivered to the brain cells of normal rats in the form of small amounts of L-p-boronophenylalanine (BPA) using the CSF administration method. In the study described here, we carried out experiments with brain tumor model rats to demonstrate the usefulness of the CSF administration method for BNCT. We first investigated the boron concentration of the brain cells every 60 min after BPA administration into the lateral ventricle of normal rats. Second, we measured and compared the boron concentration in the melanoma model rats after administering boron via either the CSF administration method or the intravenous (IV) administration method, with estimation of the T/N ratio. Our results revealed that boron injected by the CSF administration method was excreted quickly from normal cells, resulting in a high T/N ratio compared to that of IV administration. In addition, the CSF administration method resulted in high boron accumulation in tumor cells. In conclusion, we found that using our developed CSF administration method results in more selective delivery of boron to the brain tumor compared with the IV administration method.

## 1. Introduction

**Figure 1:**
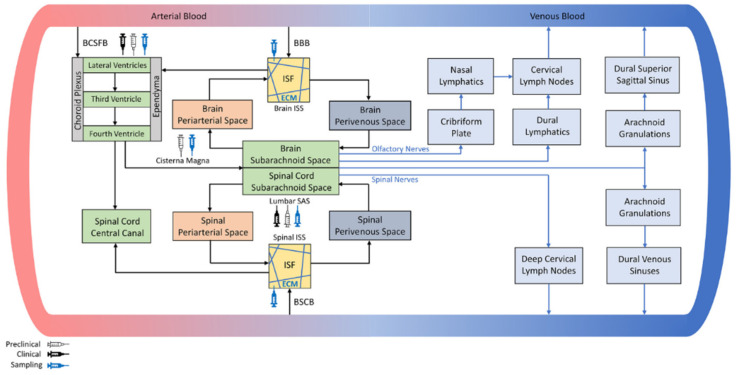
Schematic representation of the CSF net flow within the brain and spine [6].

It has recently been recognized that clinicians cannot rely on only one modality to solve the drug delivery challenges they face when treating brain tumors. Some drug delivery systems that bypass the blood–brain barrier (BBB) have been developed for brain tumor therapy, in addition to advancements in molecular engineering geared at allowing active transport across the BBB [1], the development of antibody–drug conjugates (ADCs) designed to treat brain tumors, and the transient opening of the BBB using ultrasound, to name a few examples. Systems for delivery include intranasal delivery [2,3], convection-enhanced delivery (CED) [4,5], and drug administration by injection into the cerebrospinal fluid (CSF) (intra-CSF delivery), among others. At present, CSF circulation is especially receiving increasing recognition as a feasible path to deliver certain drugs deeper into brain tissues [6]. The BBB has a crucial role in ensuring normal brain function by selectively limiting the transport of substances, such as pathogens and toxins, from entering the blood circulation. However, this function often prevents drugs from reaching brain cells. CSF is primarily produced by the choroid plexuses and flows from the lateral to the third and fourth ventricles before it enters the subarachnoid space surrounding the brain and spinal cord and, finally, drains into the veins via several pathways, as summarized in Figure 1 by G. N. Kouzehgarani et al. [6]. Administration via CSF circulation to achieve more efficient drug delivery to the brain has been attempted for over 40 years [7]. In recent years, a deeper understanding of CSF, including CSF circulation and CSF clearance pathways [8], supports the recognition of using CSF circulation as a promising route of administration. CSF administration is currently the most widely practiced approach that can bypass the BBB for drug delivery to the brain [9,10,11,12,13,14].

One of the challenges associated with boron neutron capture therapy (BNCT) [15,16] is the development of a system to efficiently deliver boron, especially to brain tumors, in the form of boron drugs, such as L-p-boronophenylalanine (BPA) and disodium mercaptoundecahydrododecaborate (BSH). In practice, the boron (^10^B) concentration in tumor cells should exceed 25 μg/g, and the ratios of boron concentrations in the tumor cells to blood (T/B) and in the tumor cells to normal cells (T/N) should be higher than 2.5 [17]. The development of boron drugs that meet the above requirements, except BPA and BSH, has been in progress. However, the addition of large numbers of modifying groups sometimes cause side effects in the human body, even though boron itself is only slightly harmful. A clinical trial of BNCT using boron modified with, for example, a porphyrin compound, was abandoned because of toxicity in the case of injections of a therapeutic dose, though the boron compound administration resulted in a high T/N ratio [18]. Unfortunately, this associated toxicity could prevent the development of new boron drugs for BNCT. Furthermore, a high boron concentration in normal cells could result in radiation exposure for patients undergoing BNCT. Thus, achieving high boron accumulation in tumor cells together with rapid boron excretion from normal cells using existing drugs (BPA and BSH) is required for improving the therapeutic efficacy [19].

Our laboratory has been developing a system for boron delivery to brain cells via CSF, which we call the “boron CSF administration method”. In our previous study, we found that use of the boron CSF administration method resulted in a level of a boron accumulation in the brain cells of normal rats that was equal to that achieved using the intravenous (IV) administration method, even though the dose of BPA was quite low (around 1/90 of the BPA dose used in the IV administration method) [20]. This result suggests that compared to the IV administration method, the method we are developing has the potential to reduce the economic and physical burden for patients with brain tumors. It is likely to become an efficient administration method for boron drugs, including BPA, which is a well-known boron carrier that can cross the BBB via active transporters. Additionally, the realization of the method could promote the clinical redevelopment of some boron compounds that were previously abandoned because of their toxicity.

In this study, we carried out experiments using a brain tumor model rat to demonstrate the usefulness of the boron CSF administration method for BNCT. To be feasible, boron accumulation in normal cells should be much lower than in tumor cells. Therefore, we first investigated the boron concentration in brain cells every 60 min after BPA administration into the lateral ventricle of normal rats to estimate the time taken for boron excretion from normal brain cells. Second, we measured the boron concentrations separately in brain tumor cells and normal cells in the melanoma model rats after administering BPA into the lateral ventricle (CSF administration method) or blood vessel (IV administration method) in order to estimate the T/N ratio. Then, based on the previously estimated excretion time from the normal brain, we set the time period required for the boron concentration in the normal cells to decrease as much as possible after the end of the infusion to the CSF.

## 2. Materials and Methods

### 2.1. Excretion Time Profile of Boron Based on Concentration in the Brain Cells of Normal Rats

We measured boron concentrations in normal brain cells every 60 min after administering BPA for 60 min into the lateral ventricles (CSF administration method) or blood vessels (IV administration method) of normal rats to estimate the time for boron excretion from normal brain tissue. We then compared the time profiles of the boron concentrations of the CSF and IV administration methods.

The protocol of the experiments in Section 2.1 and Section 2.2 was approved by the Animal Care and Use Committee for Osaka University (approval number: 2019-1-2).

This study was conducted with 8-week-old, 250~270 g, male Slc:SD rats (CLEA Japan, Inc. Tokyo, Japan). 4-Borono-L-phenylalanine (Sigma–Aldrich Corp. Saint Louis, MO, USA) was prepared as a fructose complex to increase its water solubility (20 mg/mL BPA). The boron concentration in this solution was 1067 ppm. This was determined using inductively coupled plasma atomic emission spectroscopy (ICP–AES) based on a boron standard solution [21].

Anesthesia was performed by inhalation of 3% isoflurane at an adjustable flow rate of 1.5 L/min and intramuscular injection of ravonal, 0.15 mg/kg. The rats were prepared for stereotaxic brain operation using a stereotaxic instrument (SR-5R-HT, Narishige International Ltd., Tokyo, Japan), and a guide cannula (C313G, Bio Research Center Co., Ltd. Aichi, Japan) was introduced into the lateral ventricle (at 0.8 mm caudal and 7 mm ventral to the rat brain bregma). BPA was administered at 4.0 mg/kg to twelve rats via a cannula for a period of 60 min, as summarized in Table 1. Brain samples were collected from three rats at 0, 60, 120, and 180 min after the end of the infusion. The obtained brain samples were divided into two parts; one located near the lateral ventricle (Brain Part I) and the other away from the lateral ventricle (Brain Part II). Just before sacrifice, the CSF samples were also obtained by cisternal puncture to ensure the BPA was delivered to the CSF. To compare the administration methods, 350 mg/kg of BPA was administered to eighteen rats via the cervical vein. At 0, 60, 120, 180, 240, and 300 min after the infusion ended, three rats from each time point were sacrificed, and the brain tissues were promptly obtained. These collected samples were kept in a freezer until the boron concentrations were measured with ICP–AES (ICPS-8100, Shimadzu Corp. Kyoto, Japan).

### 2.2. Boron Uptake into Brain Tumor Cells and T/N Ratio by the CSF and IV Administration Methods

A total of 24 mg/kg of BPA was administered to five rats with brain tumors through the lateral ventricle to demonstrate the clinical usefulness of the CSF administration method for brain tumors. The brain samples were collected at 120 min after the end of infusion, at which time almost all of the boron was excreted from normal cells, as described in Section 2.1. For comparison, two tumor brain samples were collected at 240 min after administering 350 mg/kg of BPA to two rats via the cervical vein.

B16F10 melanoma model rats: The animals were eight-week-old male Slc:SD rats weighing approximately 270~300 g. The B16F10 tumor cells were provided by Tohoku University and the implantation into the rat brains was conducted by CLEA Japan, Inc. The B16F10 cells adjusted to 1.0 × 10^4^ cells/3 μL were injected at a rate of 1 μL/min (for 3 min) at 3 mm right lateral, 0 mm caudal, and 4.5 mm ventral to the rat brain bregma. The rats with brain tumors were supplied for the present experiment 9 days after implantation.

The anesthesia and brain operations were performed in the same way as in the experiment in Section 2.1. As summarized in Table 2, BPA was administered to five rats via the lateral ventricle cannula at a rate of 4.0 mg/kg/h for 360 min. At 120 min after the infusion ended, their entire brains were promptly collected and preserved with isopentane solution. At 60 min after the start of infusion, the CSF was also collected for verifying BPA delivery. For comparison, 350 mg/kg of BPA was administered to two rats via the cervical vein for 60 min. At 240 min after the infusion ended, the brain tissue of each rat was promptly obtained and preserved with isopentane solution. All the brain samples were divided into the tumor and normal tissues, and the boron concentration of each tissue was measured with ICP–AES.

### 2.3. Proof of Proper Ventricular Injection

**Figure 2:**
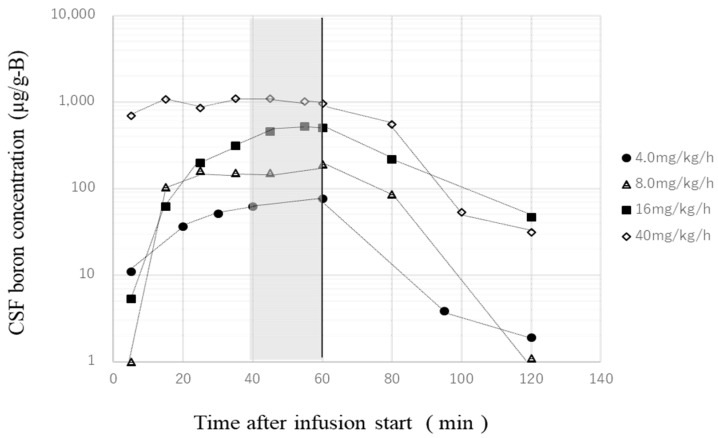
Boron concentration–time profile in CSF.

**Figure 3:**
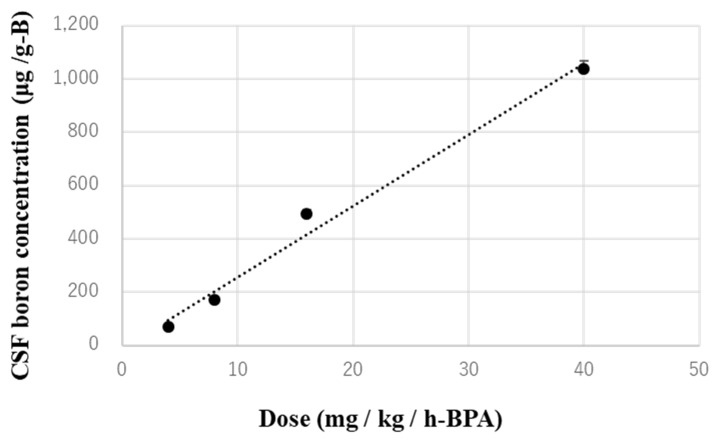
Average boron concentration in CSF from 40 to 60 min after starting infusion for each dose. The dotted line is the fitting curve of the measured average boron concentrations.

Previously, we obtained a CSF concentration–time profile during, and 60 min after, the administration of a variety of BPA doses into the lateral ventricle of normal rats, as shown in Figure 2. These CSF samples were collected by cisternal puncture every 10–20 min, and the concentrations of boron were measured using ICP–AES. Figure 3 shows the average boron concentration in the CSF from 40 to 60 min for each dose shown in Figure 2. One can see that the boron concentration in the CSF is in proportion to the dose. All of the brain samples were verified by the trajectory with the slices to ensure the BPA had been properly injected into the CSF. In this study, the results were verified by confirming that the boron concentrations achieved in the CSF were almost equal to the boron concentration on the time profile (Figure 3). Therefore, we collected the CSF sample only once, at 60 min after the start of the BPA infusion in every experiment, and confirmed that the samples met the conditions above.

## 3. Results

### 3.1. Excretion Time Profile Based on Boron Concentration in the Brain Cells of Normal Rats

**Figure 4:**
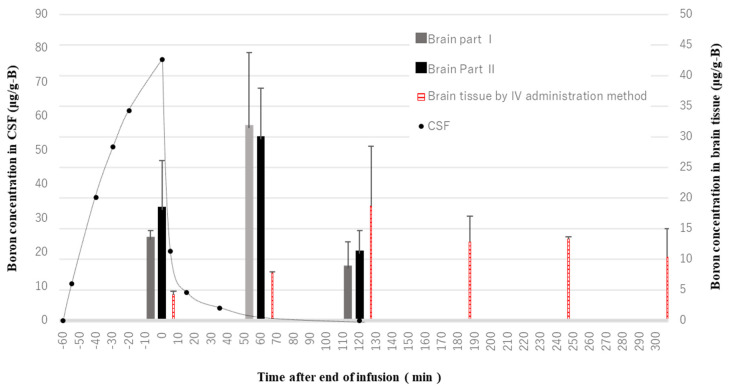
Excretion time profile of boron concentration in brain cells of normal rats. The end of infusion is 0 min.

Figure 4 shows the boron concentrations in the brain cells after administering BPA into the lateral ventricles and blood vessels of normal rats. In the case of the CSF administration method, the brain boron concentration time-to-peak was, surprisingly, found not to be immediate, but rather 60 min after the end of the infusion to the lateral ventricle, whereas the boron concentration in the CSF decreased promptly after the end of infusion. After 180 min, the boron was no longer detected. There seems to have been almost no difference in the boron concentration behavior between Brain Part I and Brain Part II. In addition, the brain boron concentration time-to-peak after the IV administration (at 120 min) occurred after that of the CSF administration (at 60 min). Furthermore, the boron by IV administration tended to be slowly excreted from the normal brain cells and still remained in the brain cells 300 min later.

### 3.2. Boron Uptake into Brain Tumor Cells and T/N Ratio by the CSF and IV Administration Methods

Although individual differences were observed, the boron concentrations in the tumor cells following the CSF administration method tended to be higher than in normal cells, as shown in Table 3. In addition, case Nos. 3–5 had high T/N ratios, meeting the conditions of the guideline that the T/N ratio should be more than 2.5, as described in Section 1. The results are comparable to the results of the IV administration method, even though the dose of BPA was quite low, that is, around 1/10 of the BPA dose used in the IV administration method.

## 4. Discussion

In this study, we investigated the boron concentration in brain tumor cells and the T/N ratio in brain tumor model rats after the administration of BPA into the lateral ventricle to demonstrate the usefulness of the boron CSF administration method for BNCT. BPA, which is one of the boron-containing delivery agents available for BNCT, is actively uptaken into cells through the L-type amino acid transporter (LAT1), which is responsible for the transport of large neutral amino acids. LAT1 overexpression is observed in a variety of tumor cells, and, thus, BPA is well-suited as a carrier for transporting boron into the tumor cells in BNCT [22]. Fortunately, since slight LAT1 expression is observed at the BBB [23], BPA is now commonly administered via the blood vessels for BNCT. Recently, a neuro-oncology study suggested that approaches less dependent on the blood circulatory system are required as an option for drug delivery in brain tumor therapy [24]. We hypothesized that harnessing CSF circulation for BPA was likely to be an efficient method for administering boron drugs in BNCT, even though the BBB is slightly permeable to BPA.

### 4.1. T/N Ratio with Tumor Model Rats by the CSF Administration Method

As shown in Table 3, some cases using the CSF administration method showed a good T/N ratio. It is important to understand the CSF clearance pathways in order to understand the behavior of boron in the brain. As can be seen in Figure 1, it is now recognized that there are several CSF clearance pathways. The idea of a glia-lymph or “glymphatic” system for waste clearance from the brain has been developed over the last five years [8]. It is now known that the glymphatic system is a major CSF clearance pathway with bulk flow via the cranial and spinal nerves, although the route by which CSF drains directly into the blood was considered the main outflux of CSF until recently [25]. Although all the details of the CSF clearance pathways have not yet been clarified [26], the idea of a glia-lymph or “glymphatic” system is likely to support our present result of a high T/N ratio by the CSF administration method, as described below.

From the results of our previous study, in the case of the IV administration method of BPA, there was a time lag between the times-to-peak of the brain and blood concentrations in normal rats [20]. A similar result was also reported in humans, namely that there was a time lag observed due to the existence of LAT1 in the BBB [27]. On the other hand, there have been no reports of attempts to investigate the time profile of boron concentrations in brain cells after administering boron drugs into the CSF for BNCT. Therefore, we first investigated the boron concentration of brain cells every 60 min after the administration of BPA into the lateral ventricle of normal rats to estimate the boron excretion time from normal brains. Firstly, the boron concentrations in the CSF were found to decrease promptly after the end of the infusion, as seen in Figure 4. The boron concentrations in the CSF can be partly explained by the fact that elimination of the drug from the CSF occurs mainly by bulk flow, as shown for cytosine arabinoside (ara-C) [28]. Secondly, the boron concentration peak in normal cells following the CSF administration method was observed at 1 h after showing the maximum concentration in the CSF. Another 2 h later, the boron was excreted smoothly from normal cells compared to that following IV administration method. This suggests that the active excretion from normal cells caused by bulk flow resulted in a high T/N ratio and, thus, CSF administration could deliver boron to brain tumors more selectively than IV administration.

### 4.2. Boron Uptake into Tumor Cells of Rats by the CSF Administration Method

In this study, the CSF administration method resulted in high boron accumulation in tumor cells, depending on the case, even though the dose of BPA was quite low. The drug injected into the CSF can arrive in the brain parenchyma via two mechanisms—diffusion or bulk flow through perivascular spaces [9]. If diffusion is the primary mechanism, then the drug concentration is expected to decrease logarithmically as drugs diffuse into the brain parenchyma from the CSF compartment because diffusion decreases with the square of the diffusion distance. Conversely, if bulk flow through perivascular spaces is the predominant pathway, a more uniform distribution of the drug in the brain is observed, with a minimal decline in the drug concentration within the parenchyma [9,29]. During drug delivery to the brain via the CSF, as documented in reports spanning the last 40 years, it has been indicated that diffusion is the primary mechanism for drug movement into the brain from the CSF surface [30,31,32]. Boron diffused into the brain parenchyma might have occurred within some hundreds of micrometers in our experiment, similar to previous reports. In addition, the logarithmic decline in the drug concentration, depending on the distance, could generate the variance in the boron concentrations observed between cases, as detailed in Table 3. However, the diffusion of the drug into the brain parenchyma from the CSF was recently estimated to be much slower than the rapid CSF elimination from the cranium because diffusion decreases in proportion to the square of the diffusion distance [8]. Moreover, the exact contribution of each process was proposed to be dependent on both the anatomical region and the size of the molecule [33].

The results of our study indicate that the boron concentration in brain cells resulting from injection of a very low dose of boron via CSF is similar to that observed for IV administration. Figure 4 also shows that this tendency does not depend on the location in the brain because the resulting boron concentrations in the Brain Parts I and II show very similar results. This clearly indicates that the boron spreads around throughout the entire brain tissue within 1 h of administration to the CSF. The mechanism of transportation from the CSF to the brain parenchyma remains controversial. We finally conclude that the results in this study imply that boron injected via the CSF arrives directly into the brain parenchyma with bulk flow through perivascular spaces rather than via diffusion. In addition, some of the boron in the CSF may rapidly drain into the peripheral blood and then re-enter the brain across the BBB, similar to the pathway taken by an intravenously injected drug [29].

### 4.3. Species Differences in Transporter Expression in the BBB and Boron Uptake into Brain Tumors

It was disappointing that the boron concentrations in the tumor cells by the IV administration method in this experiment were unexpectedly low. We expected the boron concentrations in the tumor cells of rats to be much higher than the guidelines for humans because it was reported that the amount of LAT1 protein in the blood–brain barrier, investigated by quantitative targeted absolute proteomics (QTAP), showed significant differences between rodents and primates [34], indicating that the protein amounts in humans are one-fifth of what is found in Slc:SD rats. It has not yet been revealed how the present CSF administration method could be applied to clinical treatment. However, at least in this study, the boron concentrations in the tumor cells achieved by the CSF administration method were at almost the same level as, or in some cases higher than, the boron concentrations following the IV administration method, even though the dose of BPA in the CSF administration method was quite low. Obviously, there were differences observed between the transplantable and spontaneous tumor models at the level of a molecule around the tumor tissue. With the help of veterinary medicine, we hope that research will continue on animals with spontaneous tumors.

### 4.4. Future of the CSF Administration Method

Intra-CSF delivery in animals is performed mainly via three routes to approach the brain—intracerebroventricular (ICV), intrathecal (IT; e.g., lumbar puncture), and intra-cisterna magna (ICM). In this study, we selected the ICV route for delivery of BPA into the CSF of small animals. However, the clinical application of this route in humans, which is near vital structures of the brain, might be difficult because of the risk. For clinical application in humans, we expect to administer BPA by lumbar puncture in the future, as this technique is common and the safest for patients.

## 5. Conclusions

In this study, we carried out experiments with brain tumor model rats to demonstrate the usefulness of the CSF administration method, developed by us for BNCT. As a result, we found that the boron concentration behavior clearly differed when comparing the boron CSF and IV administration methods. Compared to the IV administration method, the boron injected by the CSF administration method tended to accumulate quickly and be excreted smoothly from normal cells. Consequently, the CSF administration method resulted in a high T/N ratio. In addition, the CSF administration method resulted in high boron accumulation in tumor cells, depending on the case, even though the dose of BPA was quite low. In conclusion, this approach is indeed promising, as clinicians can inject lower doses of boron drugs into the patients, thus reducing the potential side effects of excessive amounts of the drug and decreasing the cost of the drug. We hope that the boron CSF administration method will be promoted for BNCT of brain tumors in the future.

## Figures and Tables

**Table 1:** Experimental conditions of BPA uptake into brain cells administered in normal rats using either the boron CSF administration method or IV administration method.

	CSF Administration Method	IV Administration Method
Infusion rate (mg/kg/h)	4	350
Number of animals	3 for each sampling time	3 for each sampling time
Infusion time (min)	60	60
Collected sample	CSF, Brain Part I, Brain Part II	CSF, Brain
Sampling time (min) after end of infusion	60, 120, 180, and 240 min	60, 120, 180, 240, 300, and 360 min

**Table 2:** Experimental conditions of boron uptake into brain cells by boron CSF administration method and IV administration method with BPA in melanoma model rats.

	CSF Administration Method	IV Administration Method
Infusion rate (mg/kg/h)	4	350
Animal case number	Nos. 1–5	Nos. 6 and 7
Infusion time (min)	360	60
Collected sample	CSF, tumor cell, normal cell	CSF, tumor cell, normal cell
Sampling time (min) after end of infusion	120 min	240 min

**Table 3:** Results of boron uptake into normal brain cells and tumor cells by the boron CSF administration method (Nos. 1–5) and by the IV administration method (Nos. 6 and 7) in B16F10 melanoma model rats.

Animal Case Number	Boron Concentration in Tumor Cells (µg/g)	Boron Concentration in Normal Cells (µg/g)	T/N Ratio
No. 1	23.7	13.2	1.8
No. 2	9.4	8.1	1.2
No. 3	12.4	3.7	3.4
No. 4	62.1	25.7	2.4
No. 5	19.5	4.4	4.4
No. 6	33.2	9.5	2.6
No. 7	10.2	6.3	1.3

## Data Availability

Not applicable.
